# Mobile Phones: A Possible Vehicle of Bacterial Transmission in a Higher Learning Institution in Malaysia

**DOI:** 10.21315/mjms2020.27.2.15

**Published:** 2020-04-30

**Authors:** Nur ‘Ain Hikmah, Tengku Shahrul Anuar

**Affiliations:** 1 Centre of Medical Laboratory Technology, Faculty of Health Sciences, Universiti Teknologi MARA, Selangor, Malaysia; 2 Integrative Pharmacogenomics Institute, Universiti Teknologi MARA, Selangor, Malaysia

**Keywords:** bacteria, colonisation, mobile phone, staphylococci, university

## Abstract

**Background:**

Mobile phones (MPs) have become one of the most indispensable accessories in social and professional life. Though they offer plenty of benefits, MPs are prolific breeding grounds for infectious pathogens in communities. Thus, the aim of this study was to identify the prevalence of bacterial contamination and determine antimicrobial susceptibility pattern of *Staphylococcus aureus* (*S. aureus*) from MPs.

**Methods:**

A cross-sectional study was conducted from March to July 2019 on 126 students and 37 laboratory staff/clinical instructors’ MPs from the Faculty of Health Sciences, Universiti Teknologi MARA, Malaysia by a simple random sampling technique. Along with the questionnaire, a swab sample from each participant’s MPs was collected and transported to the microbiology laboratory for bacterial culture as per standard microbiological procedures and antimicrobial susceptibility test by the disc diffusion technique. Data were analysed by the Statistical Package for Social Sciences Programme version 24.

**Results:**

All of the tested MPs were contaminated with either single or mix bacterial agents. *Bacillus* spp. (74.8%), coagulase-negative staphylococci (CoNS; 47.9%) and *S. aureus* (20.9%) were the most predominant bacterial isolates, whilst the least isolate was *Proteus vulgaris* (*P. vulgaris*) (2.5%). Oxacillin resistance was seen in 5.9% of *S. aureus* isolate. A comparison of bacteria type and frequency among gender showed a significant difference with *P. vulgaris* (*P* = 0.003) and among profession showed a significant difference with *S. aureus* (*P* = 0.004).

**Conclusion:**

The present study indicates that MPs can serve as a vector for both pathogenic and non-pathogenic organisms. Therefore, full guidelines about restricting the use of MPs in laboratory environments, hand hygiene and frequent decontamination of MPs are recommended to limit the risk of cross-contamination and healthcare-associated infections caused by MPs.

## Introduction

A mobile phone (MP) is a long-range, portable electronic device for personal telecommunications over long distances. With recent advances in the source of information and social media apps, MPs have become an indispensable accessory in social and professional life (1). Over the last few years, the role of technology has tremendously increased to facilitate a student’s learning process as the future of higher education seems to be technology driven (2). In Malaysia, the use of technological devices and advancements in Education 4.0 is expected to present significant advantages in higher learning institutions. With new technologies such as paper-thin smartphones, artificial intelligence and QR-codes, students will have more time to learn and acquire new skills (3). However, one of the most common concerns regarding heavy use of MPs is that they can act as a vehicle for transmitting pathogenic bacteria and other microorganisms (4).

Due to the advancement and benefits of MPs, their hazard to human health is often overlooked. It has been reported that a MP can harbour more microorganism than a man’s lavatory seat, the sole of a shoe or the door handle (5). The combination of constant handling and the heat generated by phone as well as sweat from hands creates an optimum breeding environment for all kinds of microorganisms that are normally found on the skin (6). This could enhanced pathogen transmission and intensified the difficulty of interrupting disease spread with now growing evidence that contaminated fomites or surfaces play a key role in the spread of bacterial infections with antimicrobial resistance such as methicillin-resistant *Staphylococcus aureus* (*S. aureus*) (MRSA) (7). A study assessed the frequency and antimicrobial susceptibility pattern of bacteria from MPs of health care workers showed 17% of the isolated were resistant to commonly used antibiotics (8).

Students and laboratory staff related to health sciences majors use their MPs while performing internships at hospitals or clinical laboratories, either to access information on their field of expertise, answer calls, text messages or take pictures during their practices (9). The frequent use of MPs in a diversity of sites raises the opportunity for cross-contamination, especially if no hygienic measures and safety practices are common among them. If pathogens are present on the surface of MPs, they could be transferred to the user skin, other surfaces or foods, where survival and growth is possible. Up to this date, most of the studies dealing with MPs are mainly focused on hospital-acquired infections and transmission of nosocomial pathogen (10, 11). Limited study has examined this aspect in Malaysia with respect to employees and students in universities. Therefore, the present study was conducted with the aim to identify the prevalence of bacterial contamination of MPs used by health sciences students and laboratory staff/clinical instructors at the Universiti Teknologi MARA, Malaysia and determine antimicrobial susceptibility pattern, with special reference to MRSA.

## Methods

### Study Design and Sample Collection

The cross-sectional study was conducted at the Faculty of Health Sciences, Universiti Teknologi MARA, Puncak Alam Campus, Malaysia from March to July 2019. Puncak Alam is located 60 km away from Kuala Lumpur, the capital city of Malaysia. There are nine departments and five health centres/clinics in this faculty. In total, the faculty has 117 lecturers, 24 administration staff, 50 laboratory staff/ clinical instructors and 1,200 undergraduate students. The Research Ethics Committee of Universiti Teknologi MARA, Malaysia (reference number: REC/296/17) endorsed the research before commencing the data collection and discussions with participants. Using Epi Info version 6, based on the desire of detecting a prevalence of MP contamination of 97.5% (12), using 95% confidence level and 5% error around the expected prevalence and an alpha error of 5%, the minimum sample size was 38 MPs. A total of 163 MPs were sampled via swabbing each device; final year students (*n* = 126) and laboratory staff/clinical instructors (*n* = 37), Department of Nursing (*n* = 5), Department of Medical Imaging (*n* = 4), Department of Environmental Health and Safety (*n* = 3), Department of Medical Laboratory Technology (*n* = 5), Department of Nutrition and Dietetic (*n* = 8), Department of Occupational Therapy (*n* = 3), Department of Optometry (*n* = 5) and Department of Physiotherapy (*n* = 4), by using a simple random sampling technique. The pre-tested questionnaire was filled out by each participant and inquired about age, gender, profession and department, background technical characteristics of the MP and usage habit. Samples were collected aseptically with a plain sterile swabs (Sterilin, UK) moistened with sterile normal saline and by rolling over the exposed surfaces of the MPs. Extreme caution was applied to make sure the switches, side of the phone, ear socket, audio input and screen were sufficiently wiped given the frequency of use of these areas by the user with their fingers and hands. In the case of MPs with covers, the swab was taken from the outer surfaces of the cover, besides the screen.

### Bacterial Identification

The swabs from MPs were collected and placed immediately into peptone water within a sterile container and transported within half an hour to the Microbiology Laboratory, Department of Medical Laboratory Technology, Universiti Teknologi MARA for microbial examination as suggested by Shooriabi et al. (13). The collected samples were inoculated onto blood agar and MacConkey agar plates (Oxoid Ltd., Basingstoke, Hants, UK) according to the standard streak plate method (14). The plates were next aerobically incubated between 24 h and 48 h at 37 °C. Primary identification of bacteria was made based on colony characteristics and Gram stain reaction microscopically. Different biochemical tests like triple sugar iron agar, indole, citrate, oxidase, urease, motility, Voges-Proskauer, methyl red, mannitol, catalase and coagulase were used for further identification.

### Susceptibility Testing

Antimicrobial susceptibility testing for *S. aureus* was carried out, employing Kirby-Bauer’s disc diffusion technique following the protocols as set by the Clinical Laboratory Standards Institute (15). In brief, the pure isolate (four to five colonies) was added to a sterile tube containing 5 mL of normal saline and mixed gently until it forms a homogenous suspension. The turbidity of bacterial suspension was standardised by using 0.5 McFarland standards. A sterile cotton swab was dipped into the suspension and inoculated the bacterial suspension over the entire surface of Mueller Hinton agar (Oxoid Ltd., UK) and left at room temperature to dry for 3 min–5 min. This was followed by placing the oxacillin disk (1 μg) (Oxoid Ltd., Basingstoke, Hants, UK) onto the MH agar and further incubated for between 18 h and 24 h at 37 °C. At the end of the incubation period, the diameter zone of inhibition was measured and interpreted as susceptible, intermediate or resistant after comparison with standard guidelines (15).

### Statistical Analysis

Statistical data analysis was carried out using the SPSS software (Statistical package for the Social Sciences) for Windows version 24 (SPSS Inc., Chicago, IL, USA). Descriptive statistics such as frequency and percentage was performed on different bacterial species. A Pearson’s Chi-square test or Fisher’s exact test was used to test the different bacteria type and frequency isolated from MPs of different gender (male and female) and profession (student and laboratory staff/clinical instructor). Statistical significance level was confirmed at *P* < 0.05.

## Results

A total of 126 students and 37 laboratory staff/clinical instructors participated in the study; 39 (23.9%) males and 124 (76.1%) females, ranging 21–53 years old. The mean age of the study participants was 27.0 (± 8.53) years. Ninety-seven percent of participants interviewed owned a MP with a touch screen and 3% had keyboard phone. Other characteristics and use of MPs by students and laboratory staff/clinical instructors are shown in Table 1.

The overall prevalence of bacterial contamination amongst the swabbed phone was 100%. Majority (76%) of isolated bacterial contaminants were mixed with more than one organism, suggestive of mixed infection and morphologically different organisms. Of these bacterial isolates, Gram-positive bacteria (74.5%) were the major isolates, of these, *Bacillus* spp. accounted for 74.8%, followed by coagulase-negative staphylococci (CoNS; 47.9%) and *S. aureus* (20.9%). Amongst Gram-negative bacterial isolates, *Klebsiella pneumoniae* (*K. pneumoniae*) (17.2%) and *Enterobacter aerogenes* (*E. aerogenes*) (16%) were the main isolates (Table 2). Overall, oxacillin (1 μg) displayed higher sensitivity against *S. aureus* with only two (5.9%) isolates were found to be resistant to this antibiotic.

Bacterial isolates contaminating MPs of both students and laboratory staff/clinical instructors were observed as reflected in Table 3. A comparison of bacteria type and frequency among both groups showed a significant difference with *S. aureus* (*P* = 0.004). In addition, a comparison of bacteria type and frequency among gender showed a significant difference with *Proteus vulgaris* (*P. vulgaris*) (*P* = 0.03) (Table 4).

## Discussion

The usage of MPs by both students and laboratory staff/clinical instructors, however, raises concerns around hygiene and the ready transmission of bacteria that could cause significant health issues. Unlike fixed phones, MPs are always picked, dropped or pocketed, therefore has the potential of acquiring microbes from the handlers and the environment. The present study revealed that all MPs tested had been prone to bacterial contamination which is also supported by research conducted in Ghana and Egypt among university students and health care workers (16, 17). These studies have also reported contamination with single or multiple bacterial types on MP’s examined. Conversely, a study on MPs among college students and staff of Birendra Multiple Campus conducted by Adhikari et al. (18) in Nepal showed a lower prevalence of bacterial contamination (56%). The observed variation might be due to the difference in adherence to infection prevention or frequency of cleaning MPs, hand washing practice and awareness of public about the role of a MP in microbial transmission.

The considerably high isolation rate of *Bacillus* spp. in this study might be related to its greater colonisation ability as well as the ability of its spores to resist environmental changes and its ability to withstand dry heat and certain chemical disinfectants. Study done by Tagoe et al. (16) among university students in science based disciplines found that *Bacillus* spp. to be the dominant isolate. They are also capable to cause food poisoning and opportunistic infections in immunocompromised host. Therefore, on the basis of the present and previous studies, it can be concluded that *Bacillus* spp. may be contaminants of MPs. CoNS are a broad group of species that commensally inhabit the human skin and mucous membranes and consequently they are the second highest isolated microorganism from MPs in the current study. Although they are less virulent than *S. aureus* and almost non-pathogenic in healthy individuals, their persistence on hospital surfaces and devices (including MPs) can be the source of blood stream infections (19). The *S. aureus* isolation rate in the present study was in line with previous study conducted in India (20). This bacteria is a major component among the normal flora of the skin and nostrils. Its predominance as the bacterial contaminants on MPs maybe because it is easily discharged by numerous human activities such as sneezing, coughing and other actions involving skin contact. Furthermore, *S. aureus* is known to cause illnesses ranging from pimples and boils to pneumonia and meningitis, a scenario supported by the high population of colony isolates (21).

The existence of Gram-negative bacteria of this research indicates the probability of contaminants on MPs as mentioned earlier on faecal flora and other objects; such organisms that originate from soil, on clothing, food and/ or on the fingers and hands of users (21). In this study, *K. pneumoniae* was the fourth most dominant bacteriological pathogen found which is lesser than the research undertaken in India (19%) (20). Indeed this form of bacteria can also be found in human faeces. The occurrence of a Gram-negative rod, *E. aerogenes* and *E. coli*, an associate of the coliforms, suggests the probability of faecal contaminants on MPs. Whereas, Gram-negative sepsis often results from *P. aeruginosa, Enterobacter* spp., *Klebsiella* spp. and *E. coli* (22). It has also been reported that the endotoxin or lipopolysaccharide (LPS) produced by associates of this cluster has been noted as a primary initiator of the clinical cause of toxic shock. In this study, *P. aeruginosa* isolation was found to be less (2.4%) which is comparable to the research of Chawla et al. (23) reporting the isolation of this bacterial at 2.5%. Notably, *P. aeruginosa* is primarily a nosocomial pathogen that could be transmitted through an object (i.e., surgical instruments, bedding and bracelets, etc). Whereas, *P. vulgaris* was the least bacterial contaminant found in the present study which existed as a nominal associate of healthy intestinal features of humans. Moreover, it has been isolated from human faeces and sewage which can, unfortunately, be accidentally transferred onto the skin through faecal contaminated, inanimate or animate material for instance due to improper hand washing after using the latrine (24).

The present study highlighted a significant difference of *S. aureus* colonisation of MPs used by students and laboratory staff/clinical instructors, thereby suggesting their regular exposure to the bacterial in the working environment, possibly transmitted in course of carrying out their professional duties. The aerosols created in laboratories and the contact of their MPs with the laboratory benches might also account for the slightly high prevalence rate of *S. aureus* as compared to their counterparts. Most organisms die within hours due to dehydration but *S. aureus* can survive for weeks and multiply rapidly in optimum temperatures, as MPs are kept warm in pockets, handbags and brief cases (25). This finding supports the previous study by Amala and Ejikema (26) reporting that about 87.5% of medical laboratory scientists’ MPs in the University of Port Harcourt’s Teaching Hospital, Nigeria were contaminated with *S. aureus*. A significant association between the isolation rate of *P. vulgaris* and gender of the users was also noted in this study. Latest study conducted by Mohammed et al. (27) also found that only one females’ MP (1.42%) was contaminated with this species as compared to their male counterparts (3.75%). The decreased contamination percentage in females’ MPs than in males’ may be referred to the fact that most females keep their MPs in their handbags, while males hold their MPs in their palms, laboratory coat pocket or put them on contaminated tables or other surfaces from which they may acquires additional *P. vulgaris*.

Interestingly, only two MRSA were found to be isolated from the MPs of the laboratory staff/clinical instructor in the current research. This is supported by the finding from other report conducted in Saudi Arabia (21). On the other hand, few researchers have detected a high rate of MRSA from MPs (23, 28). Indeed, this may have been a reflection due to these reports originating entirely from healthcare environments and the higher rate of antibiotic-resistant bacteria, MRSA, generally found in hospital settings (28). Furthermore, the lower rate of MRSA in this study could be due to the different study area and the techniques employed.

The present study however has several limitations. Firstly, the bacterial counts of the isolated microorganisms were not done and this may interfere with the assessment of the level of contaminating microorganisms present per square cm. Secondly, no isolation of fungal was done due to some technical problems. Therefore, further studies are recommended to identify fungal contamination on MPs owned by both students and laboratory staff/clinical instructors in order to have a better insight if MPs can also be a good carrier of pathogenic fungal microbial contaminant.

## Conclusion

All sampled MPs that were owned by the university health sciences students and laboratory employees were highly contaminated with various types of bacteria of which some are known as opportunist pathogens. This indicates the potential of the MPs as a fomite, which can result in community-acquired infections with possible public health implications. Therefore, periodic cleaning of MPs with 70% alcohol disinfectants as well as frequent hand washing should be encouraged as a means of curtailing any potential disease transmission. Besides, use of MPs in laboratory setting should also be limited to emergency calls only.

## Figures and Tables

**Table 1:** Characteristics and the usage of MPs by students and laboratory staff/clinical instructors at Faculty of Health Sciences, Universiti Teknologi MARA, Malaysia (*n* = 163)

Characteristic	Student n (%)	Laboratory staff/clinical instructor n (%)
Type of MP		
Touch screen phone	126 (100)	32 (86.5)
Keyboard phone	0 (0)	5 (13.5)
Use of cover protector	126 (100)	30 (81.1)
Cell phone use		
Calls and texting	126 (100)	37 (100)
Surf the internet	126 (100)	36 (97.3)
Play audios and/or videos	126 (100)	33 (89.2)
Take pictures and/or videos	126 (100)	37 (100)
View or download electronic documents	126 (100)	28 (75.7)
Other (access calendar, clock, GPS, play games)	126 (100)	37 (100)

**Table 2:** Number of isolates and types of bacteria isolated from MPs* of students and laboratory staff/clinical instructors at Faculty of Health Sciences, Universiti Teknologi MARA, Malaysia (March–July 2019)

Type of bacteria	Isolate *n* (%)
*Bacillus* spp.	122 (74.8)
CoNS	78 (47.9)
*S. aureus*	34 (20.9)
*K. pneumoniae*	28 (17.2)
*E. aerogenes*	26 (16)
*E. coli*	13 (8)
*P. aeruginosa*	9 (5.5)
*P. vulgaris*	4 (2.5)

Note:

* On some MPs, there were more than one isolated bacterial species

**Table 3:** Comparison of bacteria isolated from MPs of students and laboratory staff/clinical instructors at the Faculty of Health Sciences, Universiti Teknologi MARA, Malaysia

Type of bacteria	Student (%) (*n* = 126)	Laboratory staff/clinical instructor (%) (*n* = 37)	*P*-value
*Bacillus* spp.	92 (73.0)	30 (81.1)	0.320
CoNS	58 (46.0)	20 (54.1)	0.390
*S. aureus*	20 (15.9)	14 (37.8)	0.004^a^
*K. pneumoniae*	22 (17.5)	6 (16.2)	0.860
*E. aerogenes*	20 (15.9)	6 (16.2)	0.960
*E. coli*	12 (9.5)	1 (2.7)	0.301^b^
*P. aeruginosa*	6 (4.8)	3 (8.1)	0.426^b^
*P. vulgaris*	4 (3.2)	0 (0.0)	0.575^b^

Notes:

a significant association (*P* < 0.05);

b *P*-value was confirmed by Fisher’s exact test

**Table 4:** Comparison of bacteria isolated from MPs of male and female at Faculty of Health Sciences, Universiti Teknologi MARA, Malaysia

Type of bacteria	Male (%) (*n* = 39)	Female (%) (*n* = 124)	*P*-value
*Bacillus* spp.	32 (82.1)	90 (72.6.1)	0.234
CoNS	16 (41.0)	62 (50.0)	0.328
*S. aureus*	11 (28.2)	23 (18.5)	0.195
*K. pneumoniae*	10 (25.6)	18 (14.5)	0.108
*E. aerogenes*	6 (15.4)	20 (16.1)	0.912
*E. coli*	4 (10.3)	9 (7.3)	0.513^b^
*P. aeruginosa*	4 (10.3)	5 (4.0)	0.220^b^
*P. vulgaris*	4 (10.3)	0 (0.0)	0.003^a^,^b^

Notes:

a significant association (*P* < 0.05);

b *P*-value was confirmed by Fisher’s exact test
